# Dosimetric evaluation of Ethos 2.0 high‐fidelity mode for single‐isocenter SRS

**DOI:** 10.1002/acm2.70370

**Published:** 2025-11-21

**Authors:** Udbhav S. Ram, Richard A. Popple, John B. Fiveash, Katherine A. Heinzman, Yogesh Kumar, Natalie N. Viscariello, Courtney B. Stanley, Dennis N. Stanley, Carlos E. Cardenas, Joel A. Pogue

**Affiliations:** ^1^ Department of Radiation Oncology The University of Alabama at Birmingham Birmingham Alabama USA; ^2^ Department of Physics and Astronomy McMaster University Hamilton Ontario Canada

**Keywords:** adaptive radiotherapy, stereotactic radiosurgery, template‐based planning

## Abstract

**Purpose:**

This study evaluates the impact of the Ethos 2.0 high‐fidelity (HF) mode on single‐isocenter, stereotactic radiosurgery (SRS) planning workflows. By comparing different planning templates, we assess the effects of HF mode and control rings (R) on plan quality, aiming to optimize treatment for patients with brain metastases.

**Methods:**

A cohort of 45 patients with brain metastases was divided into a tuning (*n* = 15) and a validation set (*n* = 30). Four planning templates were evaluated: HFonRon, HFonRoff, HFoffRon, and HFoffRoff. Plans were generated using the Ethos Intelligent Optimization Engine (IOE v02.00.10), and all patients were prescribed 30 Gy in 5 fractions using 2 mm PTV margins. Plan quality was evaluated using target coverage (PTV V100%), normal brain dose (Brain‐PTV V30Gy), Paddick conformity index (CI), and Falloff index. Plan complexity was evaluated using total MU's on a per‐plan basis across the four templates.

**Results:**

The HFonRon template produced better quality plans, achieving significant improvements in normal tissue sparing (*p* < 0.0001), CI (*p* < 0.0001), and Falloff index (*p* < 0.0001) compared to other templates. All templates provided clinically acceptable target coverage. In the HFoffRoff configuration, 97.5 % of targets met the coverage criterion (V100% > 95%). The other setups achieved coverage in 99.2% (HFoffRon), 96.6% (HFonRoff), and 98.3% (HFonRon) of targets, respectively. Plan complexity decreased significantly (*p* < 0.0001) when enabling HF mode compared to both HF disabled templates

**Conclusions:**

His study shows that combining Ethos 2.0's high‐fidelity mode with user‐defined control rings yields superior single‐isocenter SRS plans compared with planning strategies that omit one or both features. This approach improves dose conformity, reduces normal tissue exposure, and decreases plan complexity through semi‐automated, template‐based planning. These findings suggest that HF mode can be a valuable tool in clinical practice for optimizing SRS treatments for patients with brain metastases.

## 1 INTRODUCTION

Automated treatment planning has been a field of rapid evolution in the context of radiation therapy (RT). Previously, plans required weeks to generate and validate, even with experienced clinical teams. Manual plan generation has demonstrated large variance and inconsistency in quality.^1^, ^2^, ^3^ The benefits of reduced inter‐observer variability within treatment planning systems have shown promise in improved clinical outcomes.^4^ To streamline the planning workflow and reduce variability in plan quality, computational tools that automate planning were implemented within existing treatment‐planning management systems (TPMS).^5^, ^6^

Varian's Ethos treatment‐planning management system (Varian Medical Systems, Palo Alto, CA) was designed with shareable planning templates and other automation‐focused features that streamline workflows and enhance plan quality. Although the Ethos TPMS was only introduced in 2020 and remains in its early stages, its first‐generation Intelligent Optimization Engine (IOE v1.1) has been thoroughly evaluated for both adaptive and non‐adaptive treatments.^7^, ^8^ The recent release of IOE v2.0 adds high‐fidelity (HF) mode, a new stereotactic planning option. HF mode removes optimizer constraints used in standard IOE planning, most notably, it introduces the use of a high‐resolution dose grid, allows greater dose heterogeneity within the target, and promises sharper dose fall‐off while simultaneously decreasing plan complexity.^9^ Recent studies have highlighted IOE v2's improvements;^8^, ^10^, ^11^ however, its high‐fidelity mode has yet to be explored for stereotactic radiosurgery of brain metastases. Although Ethos was not originally designed for stereotactic radiosurgery (SRS), its higher couch weight limit (505 lbs) and the new high‐fidelity planning mode in IOE v2 make it a viable option for patients who cannot be treated on other systems. Evaluating how HF mode affects target coverage and normal‐tissue sparing for brain metastases of varying size, therefore, has direct clinical importance.

The goal of this study was to develop and validate a semi‐automated treatment template that delivers clinically acceptable single‐isocenter plans for brain metastases on Ethos using HF mode. We investigate multiple planning strategies to evaluate individual contributions of HF mode and control rings, on dose optimization, and provide a comprehensive dosimetric analysis between planning strategies.

## 2 METHODS AND MATERIALS

### 2.1 Patient cohort

Forty‐five brain‐metastasis patients previously treated at our institution with linac‐based SRS were randomly selected for this IRB‐approved study (IRB‐300014635). Patients’ radiotherapy DICOM data were anonymized, then their planning CT scans and structure sets were imported into an Ethos 2.0 emulator. The vendor‐provided emulator was an in‐silico, non‐clinical environment to emulate treatment planning and adaptive treatments. Patients with extra‐cranial disease or clinical plans containing multiple isocenters were excluded. Target volumes spanned 0.14–93.4 cc (median = 2.0 cc). Thirteen cases involved a single target, 21 had two to four targets, seven contained five to 10 targets, and four presented with more than 10 targets.

### 2.2 Template creation

A total of 45 patients were included in this study, with 15 patients randomly assigned to a tuning cohort and the remaining 30 to a validation cohort. All plans were prescribed 30 Gy in 5 fractions to allow standardized comparison across a range of target sizes, including large lesions not amenable to single‐fraction SRS clinically.^12^, ^13^ Within the tuning cohort, we initially fine‐tuned a single planning template configured with high‐fidelity (HF) mode enabled and three control rings activated (inner, middle, and outer rings of 5, 5, and 20 mm, respectively). Objectives and priorities within this starting template were iteratively modified to allow the TPS to generate plans that met basic quality control metrics (acceptable coverage and hotspot). Once suitable plans were achieved with this starting template, it was duplicated without modification of objectives or priorities to create three additional configurations by toggling HF mode and ring usage. This resulted in four total templates representing all 4 combinations of HF mode and rings (HFonRon, HFonRoff, HFoffRon, and HFoffRoff), with identical priority orderings and objectives maintained across all variants. All four templates were then applied to the 15 tuning patients to verify consistent plan generation, but were not evaluated dosimetrically beyond the initial fine‐tuning. It is important to note that the decision to only fine‐tune the HFonRon template was intentional, as the goal of this study was to evaluate the effect of HF and control rings in IOE v2.0. The resulting templates were thus applied as‐is, without further testing, in the validation cohort.

Reports evaluating IOE v1 demonstrated that achieving the requisite steep dose fall‐off depended on auxiliary control‐ring structures;^10^ whether such rings remain necessary with IOE v2's new high‐fidelity mode is unknown. By comparing the four planning conditions described above, we isolate the individual and joint contributions of HF mode and control rings to plan‐quality metrics. In this tuning cohort, the inclusion and exclusion of user‐defined rings allowed us to evaluate their role systematically. Rings primarily improved coverage in smaller targets (< 5cc) within the tuning cohort, while HF mode provided the largest improvements in conformity and dose falloff.

When planning using the tuning cohort, it was observed that 2 full‐arc VMAT plans were superior to equidistant 9‐ and 12‐field IMRT plans, with faster plan generation time compared to 3‐arc plans. Thus, only 2‐arc VMAT plans were compared in the validation cohort. Plans clinically delivered at our institution used 0mm PTV margins, which were made possible via a 6‐degree‐of‐freedom (DOF) couch and highly resolved multi‐leaf collimators (2.5mm leaf width). However, the Ethos only has 3 DOF couch capabilities and is limited by its dual banked and staggered 10mm wide MLCs, supporting the use of a 2mm margin when aligning multiple targets treated with a single isocenter. Because these differing margin conventions preclude a fair dosimetric comparison, no direct analysis of clinical versus study‐generated plans was performed in this work. We therefore focused on all 4 templates across all HF and ring configurations as a standardized framework for evaluating template performance.

Clinical structures such as OARs (Brain, Eyes, Lens, Spinal Cord, Bones, Body, Optic Chiasm, Brainstem) were contoured automatically by the Ethos TPS, while the physician‐approved, clinical gross target volumes were used for PTV generation, which were confined to the body contour. For cases with more than one metastasis (∼75% of test cases), a Boolean operator was used to group individual PTV's into a single target (PTV_Total). Additionally, “Control Rings” were defined as avoidance structures and created using the derivations provided in Table 1. For plans where ring structures were not used (Roff), inner, middle, and outer control ring structures were set to report only. The example planning template in Table 1 does not reflect the de/activation of HF mode. Individual PTV priority was ordered based on target size (i.e., the smallest target receives the highest priority, the largest target receives the lowest priority within priority 2) on a per‐patient basis, so the optimizer secures adequate dose to those volumes first while preventing large targets from dominating the objective function. It is important to note the inclusion of target Dmax planning goals within our template, which was required to ensure plans met max dose (less than 175% of Rx) and average target dose (less than 150% of Rx) thresholds imposed as quality checks by the Ethos TPS (i.e., the TPS does not provide users with plans that violate these thresholds).

**TABLE 1:** Example Ethos 2.0 SRS planning template for a patient with three PTVs.

Priority	Structure	Derivation	Planning goal	Acceptable variation
**1**	Brain‐PTV_Total	Brain—PTV_Total	V5Gy ≤ 10%	V5Gy ≤ 20%
	PTV_Total	PTV1 + PTV2 + PTV3	V30Gy ≥ 100%	V30Gy ≥ 98%
**2**	PTV1 (smallest)	GTV1 + 2mm	V30Gy ≥ 100%	V30Gy ≥ 98%
	PTV2 (middle)	GTV2 + 2mm	V30Gy ≥ 100%	V30Gy ≥ 98%
	PTV3 (largest)	GTV3 + 2mm	V30Gy ≥ 100%	V30Gy ≥ 98%
**3**	Brain‐PTV_Total	Brain—PTV_Total	V30Gy ≤ 0.3cc	V30Gy ≤ 0.5cc
	Inner Control	PTV_Total (outer) + 5mm	V29.4Gy ≤ 0%	V29.4Gy ≤ 1%
	Middle Control	Inner Control (outer) + 5mm	V15Gy ≤ 0%	–
	Outer Control	Middle Control (outer) + 20mm	V12Gy ≤ 0%	–
**4**	PTV1	GTV1 + 2mm	Dmax ≤ 135%	Dmax ≤ 145%
	PTV2	GTV2 + 2mm	Dmax ≤ 135%	Dmax ≤ 145%
	PTV3	GTV3 + 2mm	Dmax ≤ 135%	Dmax ≤ 145%

Dose volume histogram (DVH) metrics were automatically extracted from all generated plans: PTV V100% (per‐target prescription coverage), Brain‐PTV V30Gy, falloff index ($PIV_{50\%}/TV$),^14^ and Paddick conformity index (CI, $T{V_{PIV}}^{2}/\;\left(TV\;\cdot \;PIV\right)$).^15^, ^16^ Here $TV_{PIV}$ is total target volume receiving the prescription isodose, TV is the total target volume, and PIV and $PIV_{50\%}$ are the total volumes receiving the prescription dose and 50% of the prescription dose, respectively. Optimization was performed using V30Gy objectives, which we found to be more consistent with existing literature and clinical practice.^17^, ^18^ To assess a statistically significant difference between templates, the two‐sided Wilcoxon paired non‐parametric test was used.^19^

## 3 RESULTS

**FIGURE 1:**
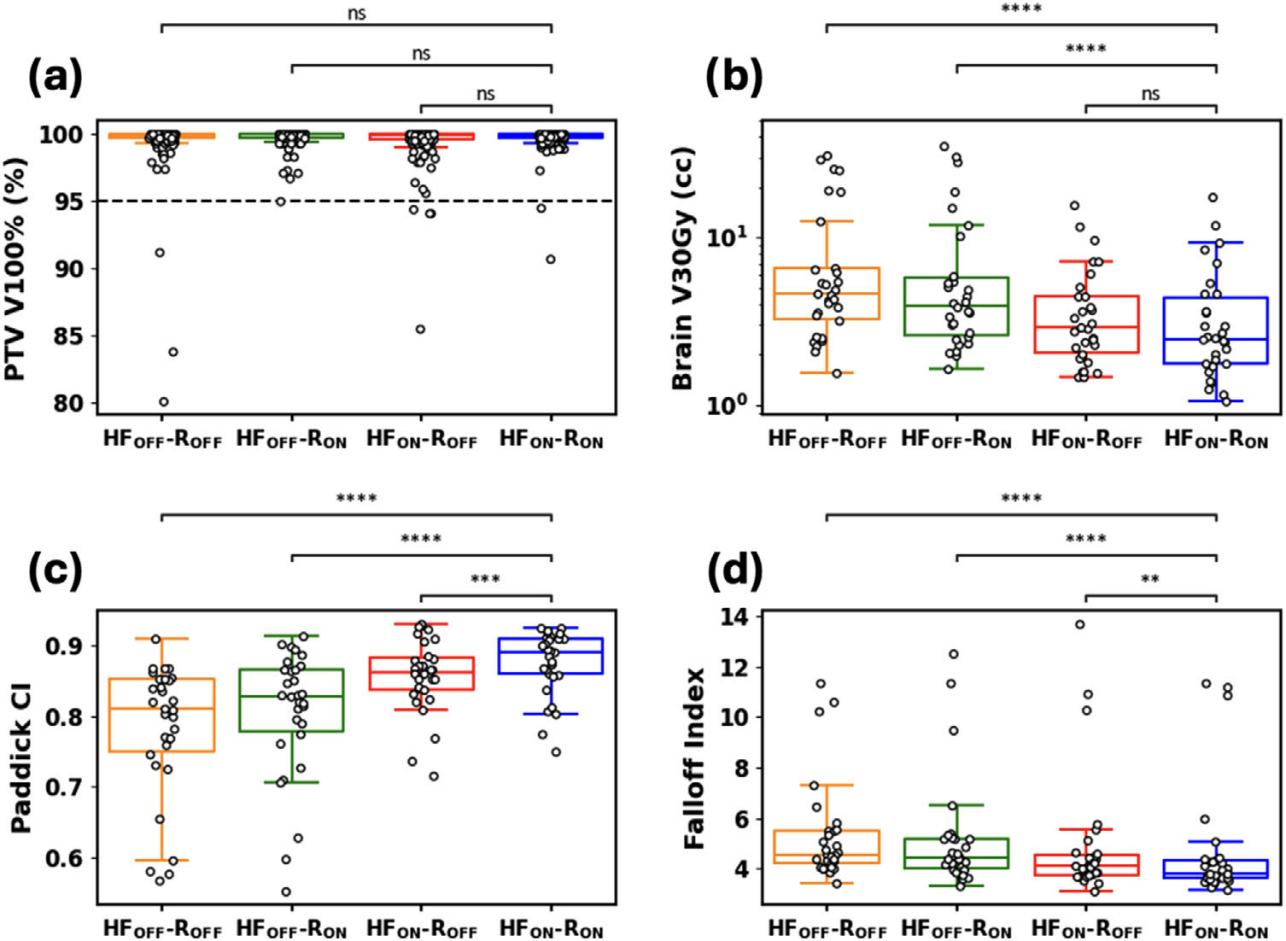
Boxplots illustrating clinical metrics across all 4 templates (a) PTV V100%, (b) Brain‐PTV V30Gy, (c) Paddick CI, and (d). Falloff Index. The two‐sided Wilcoxon paired non‐parametric test was used for difference testing, with significance values stratified as follows: ns: *p* > 0.05; *: 0.01 < *p* ≤ 0.05; **: 0.001 < *p* ≤ 0.01; ***: 0.0001 < *p* ≤ 0.001; ****: *p* ≤ 0.0001. The *X*‐axis for each shows the 4 distinct templates tested: High‐fidelity Mode (HF) and Control Ring Structures (R), ON/OFF indicating enabled/disabled, respectively. PTV V100% values are per‐target; other metrics are per‐plan. Dashed line in plot (a) indicates clinically acceptable coverage.

The boxplots in Figure 1 show comparisons of clinical metrics across the four templates.

**FIGURE 2:**
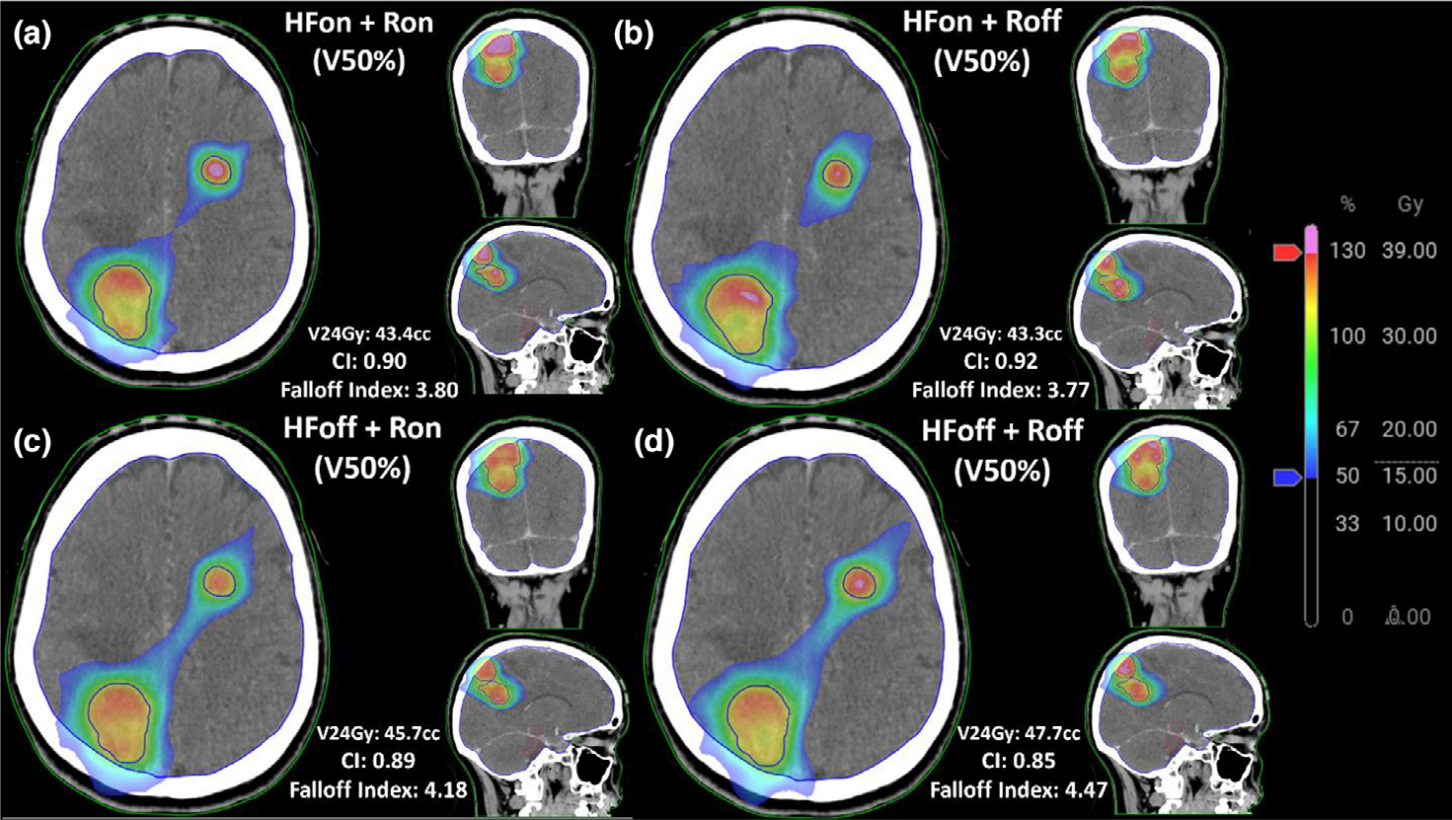
Qualitative plan comparisons on a selected patient across all 4 templates, showing increased hotspot intensity and decreased healthy brain dosage with HF mode and control rings enabled compared to all other plans.

No statistically significant difference in per‐target prescription coverage was observed between plan types, with median prescription coverage values of 99.90% for HFoffRoff, 99.97% for HFoffRon, 99.90% for HFonRoff, and 99.93% for HFonRon (Figure 1a). Median Brain V30Gy values varied significantly across plan types, with HFonRon achieving the lowest value at 2.54cc, followed by HFonRoff at 2.94cc, HFoffRon at 4.14cc, and HFoffRoff at 4.66cc (Figure 1b). Rings were not found to have a significant effect on V30Gy. CI values were significantly improved using the HFonRon template (median CI = 0.89), followed by HFonRoff (CI = 0.86), HFoffRon (CI = 0.83), and HFoffRoff (CI = 0.81, Figure 1c). Similarly, median falloff values were lowest for HFonRon (falloff index = 3.82), followed closely by HFonRoff (falloff index = 4.09), then HFoffRon (falloff index = 4.43), and HFoffRoff (falloff index = 4.55, Figure 1d). Furthermore, the qualitative improvement in falloff index and increased volume receiving dose above 130% when using HFoffRon can be visualized in Figure 2, which illustrates the dose washes between 50%–100% for all 4 templates on a representative case. There is visible bridging of the 50% isodose level when HF is not utilized, which is removed after inclusion of HF.

## 4 DISCUSSION

In this work, we assess the quality of SRS plans generated automatically from templates tuned with control rings on/off and HF on/off using the Ethos IOE v2.0. Fifteen patients were used in an iterative process to maximize plan quality, and the resulting templates were automatically applied to the remaining 30 patients in the cohort without change. Of all 120 validation plans (four plan types across the 30 patient cohort, totaling 472 targets over all 4 templates), 97.46% of targets were sufficiently covered in the HFoffRoff configuration, with 99.15%, 96.61%, and 98.31% targets achieving coverage objectives for HFoffRon, HFonRoff, and HFonRon, respectively, on a per‐target basis. The combination of control rings and HF achieved superior stereotactic plan quality to other configurations in terms of healthy brain sparing, CI, and falloff index (Figure 1). HF mode was the primary driver of conformity and sharper dose falloff, justifying the combined HFonRon template.

The goal of HF mode is specified as “allowing steeper dose” fall‐off outside targets, higher dose peaks within the target, and larger MLC openings.^9^ To achieve this, HF mode employs a single unmodifiable and hidden ring structure, which starts at 3 mm away from every target and has a thickness of 1.7 cm. In addition to the ring structure, another large normal tissue structure is created, encompassing everything outward from the outermost ring structure. These structures, which are generated internally by the IOE and not available in the planning workspace, sit between priority groups 2 and 3. In contrast, the user‐defined control rings used in this study were defined as an inner ring (5mm thickness starting from the outer edge of the PTV_Total contour), a middle ring (5mm thickness, extending from the outer edge of the inner ring), and an outer ring (20mm thickness, extending from the outer edge of the middle ring) following guidelines from our previous work.^20^, ^21^, ^22^, ^23^ The user‐defined rings are all set within the priority three group when control ring‐enabled templates were used. When both HF and user‐defined rings are enabled, the template includes the IOE‐defined ring with P2/3, in addition to the manually defined rings in P3. It is likely that narrower rings resulted in improved plan quality because a single 1.7cm ring could be considered large in the context of small (< 3 cc) SRS targets.

**FIGURE 3:**
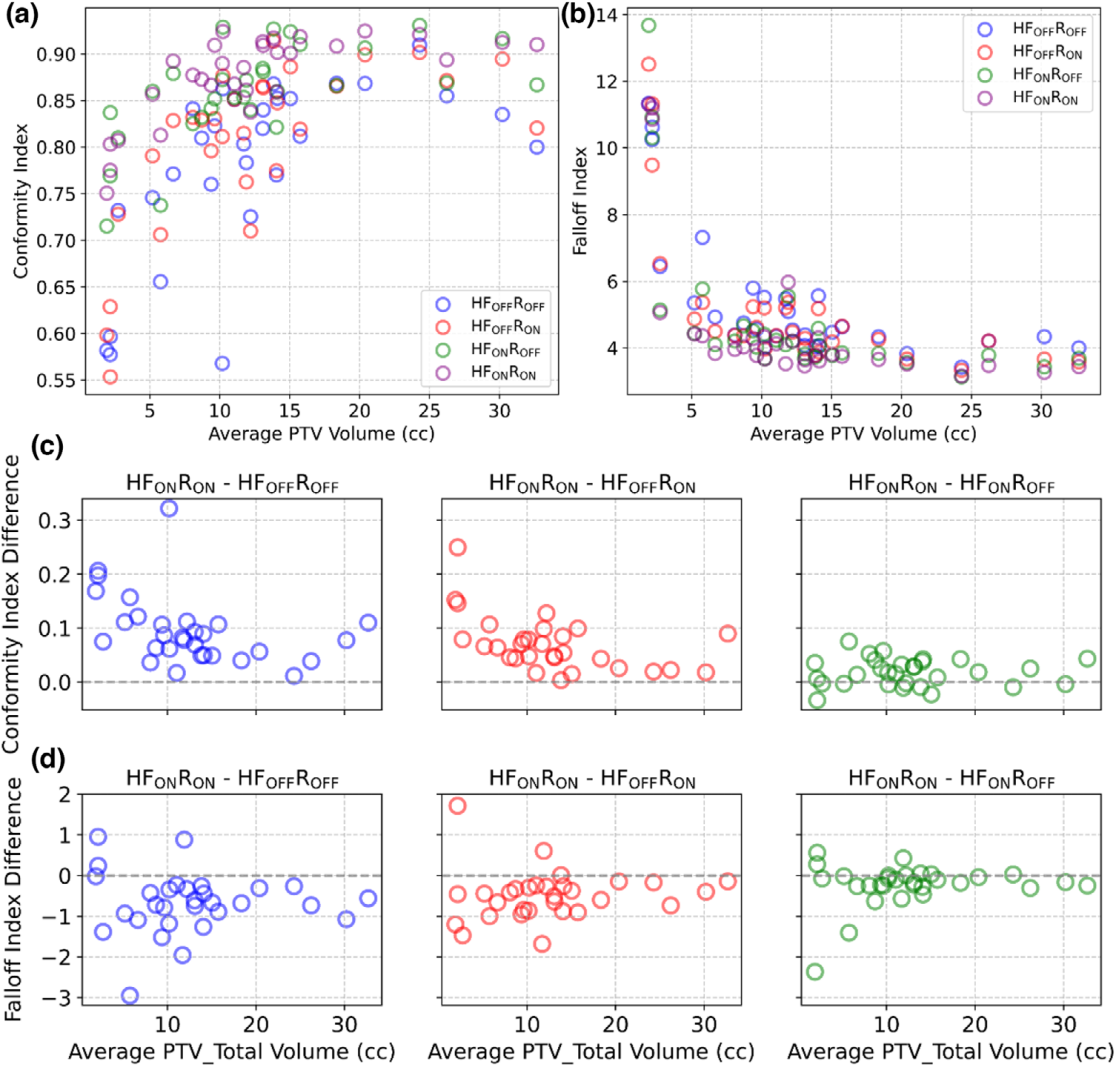
Effects of target volume on Paddick conformity and gradient indices (a) Differences in CI (b)and GI(c) between HFonRon and other templates as a function of per‐plan PTV_Total volume. All presented values are per plan.

**FIGURE 4:**
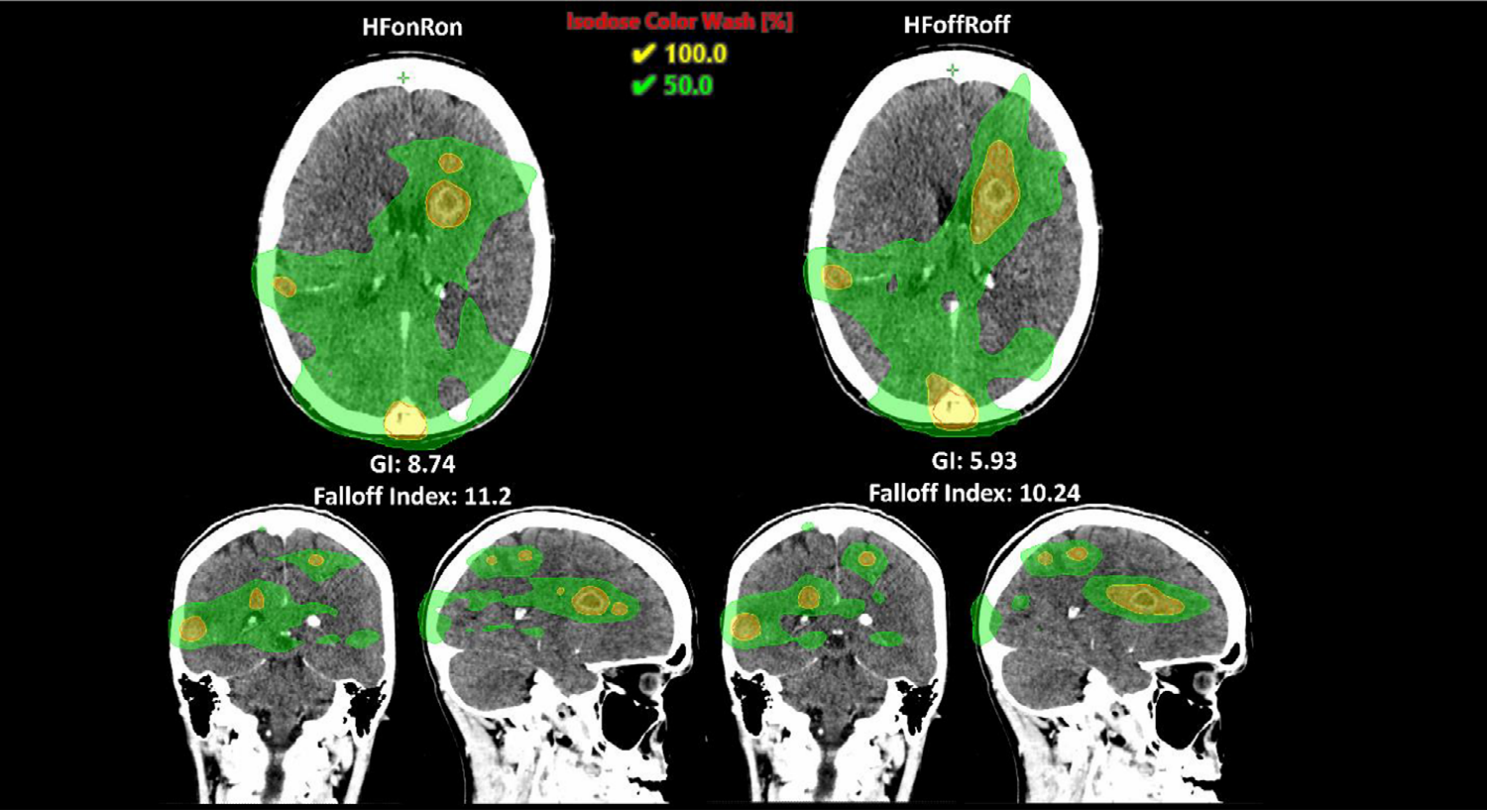
Plan comparison for sample outlier patient showing misleading GI values, and a more representative falloff index.

**FIGURE 5:**
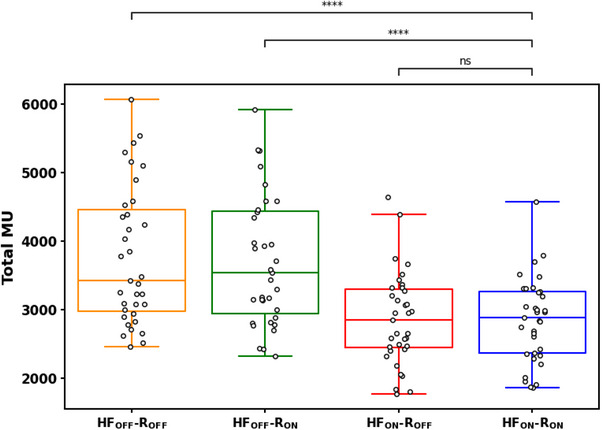
Boxplot showing plan complexity metric (MU's) across all 4 evaluated templates. The two‐sided Wilcoxon paired non‐parametric test was used for difference testing, with significance values stratified as follows: ns: *p* > 0.05; *: 0.01 < *p* ≤ 0.05; **: 0.001 < *p* ≤ 0.01; ***: 0.0001 < *p* ≤ 0.001; ****: *p* ≤ 0.0001. The *X*‐axis shows the 4 distinct templates tested: high‐fidelity mode and control ring structures (R), ON/OFF indicating enabled/disabled, respectively. All presented values are per plan.

For PTV coverage, neither HF nor rings provided statistically significant improvements across all cases. Clinically, minimizing the Brain‐PTV V30Gy dose is critical for reducing healthy brain toxicity in the five‐fraction SRS setting.^24^ In one specific patient, transitioning from the HFoffRoff template to the HFonRon template resulted in an 82% reduction in Brain‐PTV V30Gy (29.3cc to 5.3cc), which could potentially yield clinically significant toxicity differences between plan types. Furthermore, the median HFonRon falloff index was 3.82, compared to the median HFoffRoff falloff index of 4.55 (Figure 1). To add further clarity, we investigated the effect of total PTV volume on CI/falloff index, which were used as surrogates for overall stereotactic plan quality (Figure 3a,b). Our results are consistent with others, which show an inferior CI/falloff index for smaller targets.^23^, ^25^, ^26^, ^27^ As can be seen by the difference in quality metrics between HFonRon and the other three templates (Figure 3c,d), utilizing HF accounts for the largest plan quality improvement, followed by adding rings to a lesser extent. Qualitatively, we can see a similar trend in a sample patient from the validation cohort showing increased hotspot intensity and decreased healthy brain dosage with HF mode and control rings enabled compared to all other plans (Figure 2). While we did not include plans proximal to the brainstem or optics, our CI/falloff index results strongly suggest that the HFonRon template is preferred for targets proximal to dose‐limiting structures. Initially, the study planned on using the Paddick gradient index (GI) to help in quantifying plan quality. When investigating outliers, it became clear that GI was only sufficient for cases with small numbers of metastases or where the conformity did not significantly change from template to template. For cases with larger numbers of metastases or larger per‐plan conformity variation, the falloff index was found to be a more robust metric. An example case is provided below (Figure 4), where the 50% isodose was significantly larger with the HFonRon template, compared to HFoffRoff, but HFoffRoff had significantly larger 100% isodose spillage. While GI depends on both isodoses to calculate, the falloff index considers the 50% isodose and the constant volume of the target, which is a more accurate metric when the conformity varies significantly from plan to plan. In this case, we see how assessing plan quality based only on GI provides a skewed perception of the decrease in plan quality (32.2% worse GI with HFonRon) compared to the falloff index, which provides a truer representation of the difference in plan quality (8.6% worse falloff with HFonRon). Furthermore, Ethos 2.0 claims to decrease plan complexity when HF is enabled. In order to further evaluate this, a plot of total monitor units (MU) for each plan type across all validation patients is seen below in Figure 5. It shows a highly significant (*p* < 0.0001) reduction in MU (and thereby plan complexity) when enabling HF mode compared to both HF disabled templates with median MU of 3430, 3549, 2854, and 2888 for HFoffRoff, HFoffRon, HFonRoff, and HFonRon, respectively. This conflicts with existing works, which show insignificant plan complexity changes when enabling HF mode, but significantly reduced optimization time.^28^

The presented study makes several key assumptions, chief among which is the generalizability of the selected 45 patients to adequately represent the general population of patients with multiple metastases treatment courses. Since this cohort only included patients treated for intracranial disease with a single isocenter in their clinical plan, and we did not consider brainstem or optic dose constraints, the assumption of generalizability may not necessarily hold true. These inclusion criteria may lead to inferior results for use cases outside those investigated here. Further work should aim to understand the effects of proximal OARs on plan quality. Another assumption is that the contouring of other influencer structures, such as OAR's (which are automatically contoured by the Ethos TPS), is accurate and requires no modifications. Clinically, there may be additional modifications to existing contours that would affect the outcomes of plans generated using the proposed template. Another consideration is that the ordering of the PTV's from smallest to largest may not always be in line with clinical objectives, even though it was found to generate demonstrably better plans within the tuning cohort. The use of 2mm PTV margins should be evaluated on a patient‐by‐patient basis, as Ethos is limited to 3‐DOF couch correction, and patients with more targets at large separations may be more difficult to align without additional margin. Additionally, because only the HFonRon template was fine‐tuned and the other configurations were applied without re‐optimization or dosimetric recalibration, observed differences may reflect template‐calibration bias rather than the isolated effects of HF mode or ring usage, and may not represent each configuration's best achievable performance. Lastly, this study relies on plan metrics alone, and the resulting templates have not been used to generate clinical plans. Prospective studies that link dosimetric improvements to clinical endpoints would further validate the end‐to‐end clinical impacts of HF mode.

## 5 CONCLUSION

The presented study shows the first reported comparison of the impacts of the Ethos 2.0 high‐fidelity mode on single‐isocenter SRS planning workflows. Using a semi‐automated, template‐based comparative analysis, we assessed the impacts of HF mode, in conjunction with added control rings, on SRS plan quality. Prescription coverage, healthy brain sparing, Paddick CI, and falloff index results indicate that using both HF mode and user‐defined control rings results in improvements in plan quality, facilitating decreased healthy brain dose, sharper dose drop off, and reduced plan complexity compared to other planning strategies investigated.

## AUTHOR CONTRIBUTIONS

*Responsible for the tuning and evaluation of the templates as well as the research design and amalgamation of results*: Udbhav S. Ram, Dennis N. Stanley, Joel A. Pogue, and Carlos E. Cardenas. *Provided help with statistical analysis and manuscript review*: Joel A. Pogue and Carlos E. Cardenas. *Offered feedback on the manuscript*: Yogesh Kumar, Katherine Heinzman, Richard A. Popple, Natalie N. Viscariello, Courtney B. Stanley, Dennis N. Stanley, Carlos E. Cardenas, and Joel A. Pogue. *Wrote the manuscript*: Udbhav S. Ram. *Provided technical guidance, senior authorship, and support for this research*: Carlos E. Cardenas and Joel A. Pogue. All authors read and approved the final manuscript. Each author had participated sufficiently in the work to take the responsibility for appropriate portions of the content.

## CONFLICT OF INTEREST STATEMENT

The University of Alabama at Birmingham maintains research and educational agreements with Varian Medical Systems concerning the Ethos platform. Richard Popple reports that his institution, the University of Alabama at Birmingham, has product evaluation agreements and research grants with Varian Medical Systems. He also reports that he has a patent licensed by UAB Research Foundation to Varian Medical Systems, has received honoraria for presentations on behalf of Varian Medical Systems, has received a stipend to speak at Sun Nuclear meetings, and that Varian Medical Systems provides equipment to UAB as a part of a product evaluation agreement. John B. Fiveash and his institution, the University of Alabama at Birmingham, have received payment from Varian Medical Systems. Dennis N. Stanley and his institution, the University of Alabama at Birmingham, have received payment from Varian Medical Systems. Carlos E. Cardenas and his institution, the University of Alabama at Birmingham, have received payment from Varian Medical Systems.

## Data Availability

Data used in this manuscript is available upon reasonable request through correspondence with the corresponding author.
